# Commentary: Effect evaluation of *Sahtak bi Sahnak*, a Lebanese secondary school-based nutrition intervention: a cluster randomised trial

**DOI:** 10.3389/fnut.2023.1241165

**Published:** 2023-09-27

**Authors:** Abu Bakkar Siddique, Andrew W. Brown, Lilian Golzarri-Arroyo, David B. Allison

**Affiliations:** ^1^School of Public Administration, Florida Atlantic University, Boca Raton, FL, United States; ^2^Department of Biostatistics, University of Arkansas for Medical Sciences, Little Rock, AR, United States; ^3^Arkansas Children's Research Institute, Little Rock, AR, United States; ^4^Department of Epidemiology and Biostatistics, Indiana University School of Public Health-Bloomington, Bloomington, IN, United States

**Keywords:** cluster randomized control trial, error correction EC, clustering effect, nesting effect, transparency

Said et al. (1) evaluated the effectiveness of an educational school-based nutrition intervention called *Sahtak bi Sahnak* (your health on your plate) on dietary knowledge and adherence to dietary guidelines among secondary school adolescents in Lebanon. The authors concluded that the intervention was effective. However, upon closer inspection, we identified issues with the authors' statistical analysis and description of the cluster randomized controlled trial (cRCT) process. Our main concern is that the authors ignored the clustered nature of their randomization for part of their statistical analysis, including the third model (effect on unhealthy items adherence score) in **Table 2** and the entire results in Table 3 of the original paper. The authors stated, “Where the random intercept was non-significant in the multilevel analysis, a multivariate regression model was performed to test the intervention effect, adjusting for the same significant covariates.” However, this approach lacks statistical justification because a non-significant random intercept does not imply that clustering and nesting effects can be disregarded in cRCTs.

When a study aims to evaluate the effect of an intervention in which the treated unit was a group rather than an individual, the design and analysis must address the issue of clustering and nesting effects (2, 3). In Said et al. (1) study, the intervention unit was the school (a total of 16 schools, 8 assigned to the treatment group and 8 schools assigned to the control group); therefore, the statistical analysis should not be conducted at the single level such as student without accounting for clustering and nesting effects. By “clustering effects,” we mean accounting for the potential and usually positive correlation among students within the same school, which can be adjusted for by using a random effect for clusters. The authors should have also accounted for the nesting effect by adjusting the degrees of freedom or by using different approximations. Ignoring these positive correlations within clusters or the limited degrees of freedom may inflate the type I error rate (2).

The authors accounted for such clustering effects in only two places (the first and second models in **Table 2**) by applying multilevel hierarchical estimates on the dietary knowledge score and healthy items adherence score. But they ignored the clustering for all other estimates (the third model in **Table 2** and all the estimates in Table 3). Nevertheless, in these two models, the authors considered only two-level (adolescents and school), whereas their description suggests potentially more clustering levels, such as location and grades. A single-level analysis that does not account for such clustering and nesting effects for these multilevel observations produces results which, by being based on unsound analyses, are inherently unsubstantiated (2).

We also noticed that the authors did not clearly describe the process of randomization of the 16 schools while categorizing them into private vs. public, rural vs. urban, and grade 10 vs. grade 11. For the analysis to be validated, readers need to understand whether these locations and grades were clusters and whether they were appropriately randomized (4). Given the limited description in the paper, in its current form, we are unable to differentiate between whether the intervention design was random or was stratified for convenience. In a cRCT, every clustering unit should be randomized. Moreover, Said et al. (1) did not take clustering and nesting into consideration when they conducted sampling power calculations. Owing to the various levels (e.g., school types, locations, grades), the sample size and power calculations must consider the number of clusters per condition, their sizes, and their intracorrelation (5). It is also important that we highlight that Said et al. applied inappropriate chi-square and *t-*test in Table 1 to check the baseline differences between control and treated students. Usually, it is not advisable to test for baseline differences between study arms (some journals forbid it), as stated in the Consolidated Standards of Reporting Trials Guidelines, because it only tests the hypothesis of whether the randomization had been performed correctly. However, in this case, there is enough doubt about the randomization that such tests seem wise. Therefore, we eschewed the inappropriate use of the chi-square and *t-*test in Table 1 and instead used linear mixed models for continuous variables and generalized linear mixed models for multinomial outcomes adjusting the degrees of freedom for number of schools and found no significant differences in any variable of Table 1.

**Table 1:** Revised results and reproduced results from Table 2 of the original paper.

	**Reproduced results from Authors' Table 2**					**Revised results**		
	**β**	**SE**	***P*** **-value**	**95% CI**	**β**	**SE**	***P*** **-value**	**95% CI**
Dietary knowledge score	12.75	1.126	< 0.001	10.54/14.95	12.77	1.299	< 0.001	9.94/15.60
Healthy items adherence score	1.89	0.429	< 0.001	1.04/2.72	1.89	0.497	0.003	0.81/2.98
Unhealthy items adherence score	−1.43	−1.428	< 0.001	−1.82/−1.03	−1.41	0.256	< 0.001	−1.96/−0.85

To obtain the results that were originally reported in the paper, we employed the same methods described in the publication, thus achieving reproduced results. Revised results take both clustering and nesting effects into account (at least that clustering and nesting that was made explicitly clear in the article).

We thank Said et al. (1) for sharing their raw data and offering feedback to uphold the rigor of scientific research which have allowed us to reanalyze their work and present revised results in this letter (Tables 1, 2). While our reanalysis using their methods as explained in the paper generated some of their results that are close to original estimates in terms of both size of coefficients and statistical significance level, we have also failed to closely reproduce results in other cases. It is important to note that our analysis did not overturn Said et al. (1) conclusions or result in changes of formal statistical significance, and therefore no change in the paper's conclusion about the effects of the *Sahtak bi Sahnak* intervention Although the results were not qualitatively different, it is essential that we present the revised analysis using appropriate methods. Moreover, the confidence intervals were wider and the coefficient estimates differed between original and revised estimates, which has important implications particularly for research syntheses like meta-analyses. Our reanalysis assumed that there were only two levels—school and adolescents—in the intervention design. If there are more than two levels, which could not be confirmed because of the unclear description of the randomization process in the paper, we can neither support nor refute whether the *Sahtak bi Sahnak* intervention was effective in increasing dietary knowledge and adherence to dietary guidelines. Despite the qualitatively similar results, we present the corrected results because it is an ethical and professional scientific responsibility to correct any reported errors in published papers (6, 7). The statistical code used for reproducing the results and for analyzing the results with accounting for clustering and nesting (acknowledging that we could only account for the clustering and nesting that were explicitly described in the article) are available at the following link: https://doi.org/10.5281/zenodo.8030967.

**Table 2:** Revised results and reproduced results from Table 3 of the original paper.

	**Reproduced results from Authors' Table 3**					**Revised results**		
	β	**SE**	***P-*** **value**	**95% CI**	β	**SE**	***P*** **-value**	**95% CI**
**Total dietary knowledge score**								
**Grade**								
Grade 10	11.13	0.629	< 0.001	9.89/12.36	11.73	1.370	< 0.001	8.74/14.71
Grade 11	13.64	0.575	< 0.001	12.51/14.77	13.88	1.519	< 0.001	10.52/17.24
**Weight status**								
Underweight	15.39	1.283	< 0.001	12.85/17.93	15.39	1.308	< 0.001	12.39/18.39
Healthy weight	12.51	0.563	< 0.001	11.14/13.61	12.95	1.454	< 0.001	9.78/16.13
Overweight/obese	10.61	0.804	< 0.001	9.03/12.19	11.17	1.566	< 0.001	7.75/14.58
**Knowledge score at baseline**								
Low	12.21	0.436	< 0.000	11.36/13.07	12.62	1.342	< 0.000	9.69/15.55
Acceptable	9.93	6.118	0.127	−3.20/23.05	10.62	7.871	0.298	−20.13/4 1.37
**Healthy items adherence score**								
**Location**								
Urban	−1.55	0.257	< 0.000	−2.06/−1.05	−1.43	0.567	0.059	−2.95/0.08
Rural	−1.30	0.209	< 0.000	−1.71/−0.89	−1.26	0.361	0.017	−2.18/−0.34

To obtain the results that were originally reported in the paper, we employed the same methods described in the publication, thus achieving reproduced results. Revised results take clustering and nesting effects into account (at least that clustering and nesting that was made explicitly clear in the article).

## Author contributions

AS: conceptualization, data analysis, formal analysis, and writing—original draft preparation. AB, LG-A, and DA: conceptualization, review and editing, and approval of the final version. All authors have read and agreed to the published version of the manuscript.
